# Microbial *nar*-GFP cell sensors reveal oxygen limitations in highly agitated and aerated laboratory-scale fermentors

**DOI:** 10.1186/1475-2859-8-6

**Published:** 2009-01-15

**Authors:** Jose R Garcia, Hyung J Cha, Govind Rao, Mark R Marten, William E Bentley

**Affiliations:** 1 Fischell Department of Bioengineering, University of Maryland, College Park, MD 20742, USA; 2 Department of Chemical and Biomolecular Engineering, University of Maryland, College Park, MD 20742, USA; 3 Department of Chemical Engineering, Pohang University of Science and Technology, Pohang 790-784, Korea; 4 Department of Chemical and Biochemical Engineering, University of Maryland Baltimore County, Baltimore, MD 21052, USA

## Abstract

**Background:**

Small-scale microbial fermentations are often assumed to be homogeneous, and oxygen limitation due to inadequate micromixing is often overlooked as a potential problem. To assess the relative degree of micromixing, and hence propensity for oxygen limitation, a new cellular oxygen sensor has been developed. The oxygen responsive *E. coli *nitrate reductase (*nar*) promoter was used to construct an oxygen reporter plasmid (pNar-GFPuv) which allows cell-based reporting of oxygen limitation. Because there are greater than 10^9 ^cells in a fermentor, one can outfit a vessel with more than 10^9 ^sensors. Our concept was tested in high density, lab-scale (5 L), fed-batch, *E. coli *fermentations operated with varied mixing efficiency – one verses four impellers.

**Results:**

In both cases, bioreactors were maintained identically at greater than 80% dissolved oxygen (DO) during batch phase and at approximately 20% DO during fed-batch phase. Trends for glucose consumption, biomass and DO showed nearly identical behavior. However, fermentations with only one impeller showed significantly higher GFPuv expression than those with four, indicating a higher degree of fluid segregation sufficient for cellular oxygen deprivation. As the characteristic time for GFPuv expression (approx 90 min.) is much larger than that for mixing (approx 10 s), increased specific fluorescence represents an averaged effect of oxygen limitation over time and by natural extension, over space.

**Conclusion:**

Thus, the pNar-GFPuv plasmid enabled bioreactor-wide oxygen sensing in that bacterial cells served as individual recirculating sensors integrating their responses over space and time. We envision cell-based oxygen sensors may find utility in a wide variety of bioprocessing applications.

## Background

It is well known that oxygen limitations during bacterial fermentation can be deleterious to cell growth and productivity due to either diminished respiratory activity or the production of inhibitory byproducts [1]. While it's clear that low bulk oxygen concentration (either in the entire tank, or simply in particular regions of the tank) can lead to oxygen limitations, what is often not well understood is that significant oxygen limitations can occur even when the *bulk *dissolved oxygen concentration is well above the critical level. This phenomenon occurs as a result of fluid segregation, or incomplete micromixing.

The concept of fluid segregation, or micromixing, was first described nearly 50 years ago [2,3], has become a part of classical chemical-reaction engineering [4], and appears in a number of textbooks [5-7]. Yet, those conducting fermentations often think primarily in terms of macromixing, or movement of bulk fluid throughout the bioreactor. In contrast, micromixing describes mixing at the molecular scale. This can be understood when considering how a component fed to a continuous stirred tank reactor interacts with the contents of the tank. At one extreme is maximum-mixedness, where the added fluid molecules are immediately dispersed throughout the tank, and uniformly mixed with the tank contents until they leave the reactor. At the other extreme is complete segregation which occurs when the added fluid is dispersed into discrete fluid elements, or packets, which remain intact until they exit the reactor. As a result, each fluid element acts, in effect, as a miniature batch reactor. This latter case is likely to exist in many bioreactors where the size of the smallest turbulent eddy (approximately 100 microns [8]) is often an order of magnitude larger than typical cells. Dunlop and Ye [9] have eloquently compared this situation to microbes sitting in "a stagnant pool" which can be rapidly depleted of nutrient. Whether or not cells will be effected by this situation depends on the characteristic reaction time of relevant cellular processes, and the time cells spend in regions where the reactant concentration differs from the mean [10]. While extensive studies have shown that the degree of micromixing can impact chemical reactors [11], a limited number of studies have shown that it can also significantly impact bioreactors growing bacteria [12], yeast[13,14] and filamentous fungi [15,16].

This implies that assessing the degree of micromixing, and whether or not this will impact the cellular system of interest, is of great importance. In chemical reactors, this is typically done using test reactions which act as molecular probes [17-20], and a good test reaction should[19]: (i) employ simple reaction schemes in order to avoid analysis of many products, (ii) involve easy analysis of reaction products, (iii) involve reaction kinetics faster than the mixing rate, and (iv) show a high degree of sensitivity and reproducibility. To our knowledge, no biological micromixing-testing system has yet been developed that encompasses all of these characteristics.

**Figure 1:**
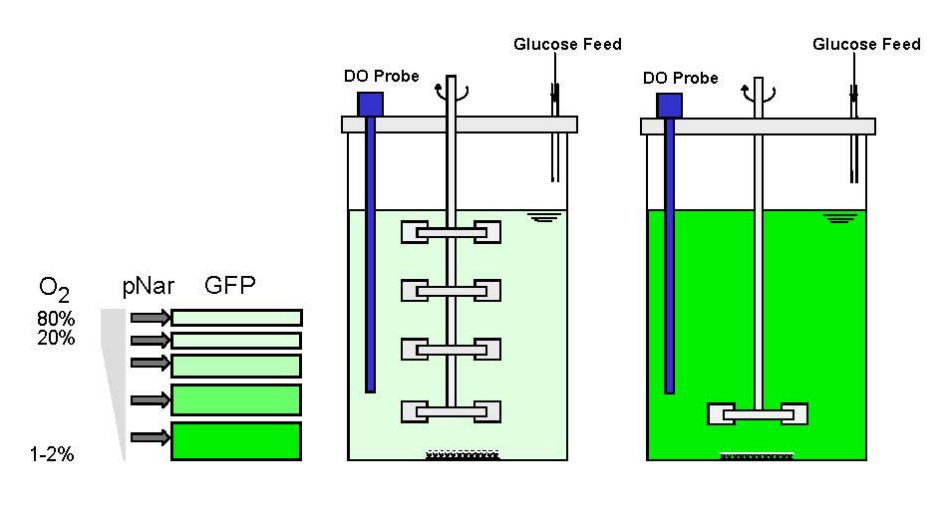
**Evaluation of oxygen-sensing cells using pNar-GFPuv construct**. Lee and coworkers demonstrated the *E. coli nar *promoter is maximally induced at microaerobic oxygen levels (1–2% of air saturation, left panel). Identical fermentors were run in parallel and in triplicate, with identical inoculums and operating conditions but with one exception: one tank was equipped with one impeller, the other with four. Our motivation was to test whether the cells would differentially express GFP as they grew and circulated around the tanks.

Our goal here was to develop bioprocess-friendly system capable of assessing the relative degree of micromixing in a bioreactor. Coincidently, we wanted to test whether oxygen limitations might be prevalent in a well-mixed laboratory scale reactor. To accomplish this, we exploit recombinant bacterial cells as 10^12^-10^15 ^continually circulating sensors that communicate the degree to which cells have experienced oxygen deprivation. In doing so, these cellular sensors provide a relative measure of the degree of micromixing present in the tank. We note that oxygen deprivation in micromixed zones is due to the mixing phenomena as well as the respiration rate of cells contained within a "fluid packet". Cellular sensors were constructed by adding the well-characterized nitrate reductase (*nar*) promoter into a pBR322-based plasmid for induction of the green fluorescent protein (GFP) reporter. Previous studies by Lee and coworkers have shown that the *E. coli nar *promoter is not significantly induced until the dissolved oxygen level drops below 20% of air saturation, and is maximally induced under microaerobic conditions (e.g., < 1–2% air saturation) [21-23]. Correspondingly, GFP is widely used as a marker for gene transcription [24-27], hence *nar*-driven GFP fluorescence indicates oxygen limitation. This concept is depicted in Figure 1, where two highly agitated (> 400 rpm) and aerated (1 vvm) lab-scale fermentors were identically run in triplicate but with different impeller geometries. Our hypothesis was that if we could set up conditions that enabled nearly identical profiles at the macroscopic level (optical density, glucose, oxygen level), the cell-based sensors might reveal subtleties in micromixing – or otherwise suggest that the cells traversed regions of sufficient and/or deprived oxygen over the time course of their growth in the fermentor. To our surprise, the cells did reveal differences in GFP fluorescence, indicative of oxygen limitation. This suggests that even in fermentors commonly assumed to be oxygen-sufficient, there are limitations perceived by the cells that, importantly, affect their gene regulation.

## Results and discussion

In order to induce varied microcirculation patterns in our fermentor, the same tank was used with two different impeller configurations – either four impellers representing a typical well-mixed flow pattern or with one impeller "hypothetically" simulating a poorly-mixed bioreactor. We carried out a series of mixing time tests with both configurations and found that in both cases 95% mixing time was less than 10 s (data not shown). This implies that both configurations, even the single impeller case, would typically be considered "well-mixed."

**Figure 2:**
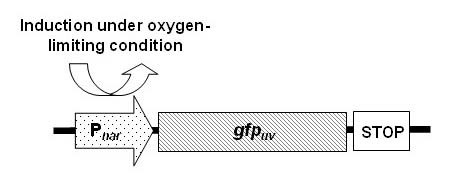
**Gene map of recombinant plasmid pNar-GFPuv**. *gfp*_*uv *_gene is regulated by *nar *(P_nar_) promoter.

**Figure 3:**
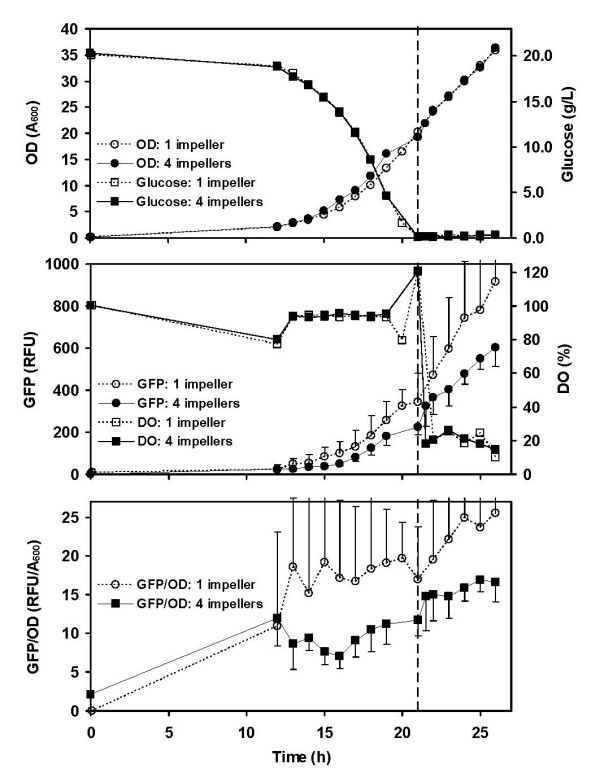
**Growth and GFP expression during fed batch fermentations with either one or four impellers (all else equal)**. (A) Biomass, measured as optical density (OD, A_600_), and glucose concentration (g/L), (B) Green fluorescent protein expression (RFU) and dissolved oxygen concentration (% air saturation) and (C) specific GFP expression (RFU/A_600_). Dashed vertical line at 21 hours shows where batch phase ended and feeding began. Error bars represent standard deviation, drawn in only one direction for clarity.

We then used these two impeller configurations to carry out triplicate fed-batch fermentations with *E. coli *harboring our pNar-GFPuv plasmid (Figure 2). As noted above, all fermentations were run batchwise until the initial glucose concentration (20 g L^-1^) was consumed to less than 1 g L^-1 ^(also marked by a rapid increase in %DO). Results are shown in Figure 3. Depicted data are the averages from three separate fermentations; the error bars denote standard deviation. We note that traditional measurements (glucose profile, OD_600_, and DO) all show nearly identical behavior. However the GFP trends (Figure 3B) show significantly higher expression when only one impeller is used versus four. This is represented even more dramatically in Figure 3C where specific GFP expression rose significantly higher with one versus four impellers.

Our findings are consistent with those reported previously for both yeast [9,10,13] and bacteria [12] which have shown a reasonably high degree of fluid segregation, and hence nutrient limitation, even in relatively small tanks. A high degree of fluid segregation implies that most oxygen mass transfer occurs in a small region of the tank near the impellers. When cells travel away from this region they do so in a "fluid packet" that may become depleted of oxygen before revisiting an impeller region. Thus, when there is only one impeller in the tank (versus four) cells will visit an impeller zone less frequently and as a result experience a relative greater degree of oxygen deprivation. This is what we see in Figure 3C. Note also that early in the fermentations, when the biomass concentration is low, there would be fewer cells in each "fluid packet." Thus it would take a longer period of time to deplete a fluid packet of oxygen. This is consistent with our finding that specific GFP expression was relatively low during the first 12 h of all fermentations. Between 10 and 20 hours, we observe relatively high DO values (> 80% air saturation) accompanying rising GFP expression. This implies oxygen mass transfer limitation occurs downstream of the gas-liquid interface, making k_L _a irrelevant in this part of the process.

We note that expression of recombinant GFP in *E. coli *requires approximately 90 min [24,28], much longer than the time required for mixing (i.e., < 10 s). Thus, there is not a direct connection between the GFP fluorescence value and instantaneous micro-DO levels. Rather, the GFP differences observed between 1 and 4 impellers in Figure 3C represent an averaged effect of oxygen limitation over time. Correspondingly, we measured cells taken from the fermentor from one sample port; hence, our sample represents a spatially averaged population. The fluorescence then indicates the integrated exposure to oxygen deprived zones. Evidence of oxygen-deprivation dependent byproduct production (e.g., formate) is another means by which poor micromixing can be ascertained[29], however uncertainty of formate degradation and ease of assay make this approach less convenient.

We note that because our cell sensors indicate a temporally and spatially integrated value of oxygen limitation, we have not carried out the converse set of experiments: intentionally manipulating oxygen levels in fermentations to report on the dynamic range of the sensing cells. That is, GFP expression changes between the initial periods of the fermentations (high DO) and later times (when there is more cell mass) and the DO can be easily controlled using conventional means (e.g., varied agitation and sparging rates). A set of DO-stat fermentations with a transition from N_2 _to air might be undertaken in future studies. Instead, the approach taken here integrates all of these dynamics yielding a single response that can be easily measured and is informative.

## Conclusion

The pNar-GFPuv construct represents a simple and convenient way to assess the relative degree of micromixing in a bioreactor, and could thus be used as a diagnostic tool to study both industrial and lab-scale fermentors, to determine their propensity for oxygen limitation. This system has the advantage that it addresses micromixing test system requirements as described by [30] and discussed in the introduction. Specifically, it employs a simple reaction scheme (i.e., only GFP is expressed and the level of green fluorescence corresponds to the degree of oxygen limitation), involves a simple analysis of only one product (i.e., measurement of whole broth fluorescence for GFP expression), involves reaction kinetics that over time show the behavior of the system (i.e., relative degree of oxygen limitation) and shows both a high degree of sensitivity and reproducibility. Perhaps more importantly, this experimental system has the advantage of being very familiar to those that would likely use it in the biotechnology industry. It involves both reactants and products likely to be similar to those already used in the bioreactor being tested. In contrast, more traditional micromixing test systems involve exotic chemistries, and as such are unlikely to be used by those in the biotech industry.

## Methods

### Cell culture conditions/strains

The plasmid pNar-GFPuv (Figure 2) was constructed as per [26], wherein the reporter gene *gfp*_*uv *_(polymerase chain reaction (PCR) amplified from the pGFPuv plasmid (Clontech)) was placed under control of *nar *promoter (PCR amplified from *E. coli *K-12 (ATCC 29425) genomic DNA using primers; forward: 5'-ccgccgagatctttgattttctatatcgcc-3' & backward: 5'-gcgcggtaccctcctgtgggagcctgtcgg-3') in a pBR322 background. *E. coli *W3110 was used in all fermentations. A high cell density culture (HCDC) medium used and was prepared according to [31]. For a 4 L batch culture, stock solutions of KH_2_PO_4 _(133 g L^-1^), (NH_4_)_2_HPO_4 _(40 g L^-1^), citric acid (170 g L^-1^), EDTA (0.84 g L^-1^) and trace elements (0.25 g CoCl_2_·6H_2_O L^-1^; 1.5 g MnCl_2_·4H_2_O L^-1^; 0.15 g CuCl_2_·4H_2_O L^-1^; 0.3 g H_3_BO_3 _L^-1^; 0.25 g Na_2_MoO_4_·2H_2_O L^-1^; 1.3 g Zn(CH_3_COO)_2_·2H_2_O L^-1^; 10 g Fe(III)citrate L^-1^) were mixed in the bioreactor with 3 L of deionized water and pH-adjusted to 6.3 with 5 M NaOH. The bioreactor was then sterilized for 60 minutes at 121°C. Stock solutions of MgSO_4_·7H_2_O (600 g L^-1^) and glucose (200 g L^-1^) were sterilized separately for 20 minutes at 121°C. Thiamine·HCl (4.5 g L^-1^) and ampicillin (1 g/10 mL) were sterilized by filtration. Once cool, the remaining solutions were added (glucose, MgSO_4_·7H_2_O, Thiamine·HCl, and ampicillin). The pH was further adjusted to 6.7 prior to inoculation using NH_4_OH (28% w/w). For preparation of the feed medium, stock solutions of glucose (420 g L^-1^), MgSO_4_·7H_2_O (600 g L^-1^) and trace elements (0.4 g CoCl_2_·6H_2_O L^-1^; 2.35 g MnCl_2_·4H_2_O L^-1^; 0.25 g CuCl_2_·4H_2_O L^-1^; 0.5 g H_3_BO_3 _L^-1^; 0.4 g Na_2_MoO_4_·2H_2_O L^-1^; 1.6 g Zn(CH_3_COO)_2_·2H_2_O L^-1^; 4 g Fe(III)citrate L^-1^) were sterilized separately and combined.

### Precultures

Primary inocula were prepared by combining 100 mL of LB media, ampicillin (100 μg L^-1^), and 1 mL of freezer stock in a shake flask, which was grown 4 hours at 37°C and 250 rpm to mid-log phase. To adapt the cells to fermentor conditions, seed cultures were prepared from the primary inocula (5% v/v) using 200 mL of HCDC media, ampicillin (100 μg L^-1^), and glucose (initially 8 g L^-1^). These seed cultures were grown for 10 hours to mid-log phase (OD_600 _= 2 – 3) at 250 rpm and 30°C.

### Batch and fed-batch fermentations

All *E. coli *fermentations were carried out in a 5 L BioFlo III fermentor (New Brunswick Scientific Co., Inc.) with a 4 L working volume. Prior to inoculation with 5% v/v seed culture, the reactor conditions were pH 6.7, 30°C, 1 vvm air flow, dissolved oxygen (OD_600_) 100%, and a 400 rpm agitation rate. Temperature was regulated at 30°C by a heating jacket and pH at 6.7 by addition of aqueous 28% NH_4_(OH). Sterile antifoam 204 (Sigma Chemical Co) was added via a syringe to control excess foaming during the fermentations. The dissolved oxygen was maintained above 80% air saturation during batch operation and at 20% air saturation during fed-batch operation. In all experiments, DO was controlled by a PID controller which manipulated the stirrer speed from 400 to 1000 rpm. To maintain oxygen levels, pure oxygen was added to obtain a 50/50% mixture of air and oxygen in the inlet air stream, typically 12 hours after the fermentor was inoculated.

### Feeding strategies

The feeding strategy was based on a stepwise increase in the glucose feed rate, which approximates the exponential feeding rate described by [32]. All fed-batch experiments began with a batch phase that lasted between 19–20 hours, during which time most of the initial glucose was consumed (20 g L^-1^). The beginning of fed-batch phase was marked by a glucose concentration below 1 g L^-1^, an increase in pH, as well as a rapid increase in %DO. The pH and temperature conditions remained constant during the fed-batch phase. Additional ampicillin (4 mL) was added at the end of the batch phase to reduce potential for plasmid-free segregants.

### Analytical methods

Optical density (OD_600_) was measured hourly using a spectrophotometer (DU 640, Beckman, Fullerton, CA). Samples were diluted with deionized water to obtain OD_600 _in the linear range (0–0.5 OD_600 _units). Glucose concentration of the supernatant was measured using a glucose analyzer (YSI Model 2700, Yellow Springs, OH). Off-line fluorescence intensity was measured using a fluorescence spectrometer (LS-50; Perkin-Elmer Ltd., Beaconsfield, Buckinghamshire, England) at excitation and emission wavelengths of 395 and 509, respectively. Whole broth samples were diluted as necessary to stay within the linear range of detection. Mixing time (95%) for different impeller configurations was determined as described previously [33]. Briefly, a small amount of alkaline solution (2 drops of 28% NH_4_OH) was added to DI water at pH ~4.0 and the change in pH was monitored with time.

## Competing interests

The authors declare that they have no competing interests.

## Authors' contributions

JRG performed fermentation experiments. HJC created the *nar*-based GFP vector. GR provided insight on GFP, its folding and detection. MRM provided insight on micromixing and manuscript preparation. WEB helped with experimental design, data interpretation, and manuscript preparation.
